# A new species of the land planarian *Issoca* sheds light on the polyphyletic status of the genus (Platyhelminthes, Tricladida, Geoplaninae)

**DOI:** 10.3897/zookeys.752.24615

**Published:** 2018-04-23

**Authors:** Ana Paula Goulart Araujo, Fernando Carbayo

**Affiliations:** 1 Laboratório de Ecologia e Evolução, Escola de Artes, Ciências e Humanidades, Universidade de São Paulo – USP, Av. Arlindo Bettio, 1000, CEP 03828-000, São Paulo, SP, Brazil; 2 Programa de Pós-Graduação em Zoologia, Departamento de Zoologia, Instituto de Biociências, Universidade de São Paulo, Rua do Matão, Trav. 14, 321, Cidade Universitária, CEP 05508-900, São Paulo, SP, Brazil

**Keywords:** Brazil, Continenticola, flatworm, histology, morphology, Neotropical region, taxonomy

## Abstract

A new species of the genus *Issoca* (Platyhelminthes, Tricladida, Geoplaninae) is described. *Issoca assanga* sp. n. presents the diagnostic features of the genus, with the exception of the relative position of the subneural parenchymal muscle layer with the cephalic retractor muscle, which are overlapped in the type species of the genus but are intersected in the new species. Rather than a polymorphic character, the relative position of these muscle layers might reflect the polyphyletic status of the genus.

## Introduction

Most Neotropical land planarians are grouped in the subfamily Geoplaninae (Platyhelminthes, Tricladida, Geoplaninae). The number of known species of this group exceeds 290 species (http://planarias.each.usp.br; accessed in 2. Apr. 2018). The genus *Issoca* Froehlich, 1955 (Geoplaninae) comprises five species, namely *I. spatulata* (Graff, 1899), *I. rezendei* (Schirch, 1929, type species of the genus), *I. jandaia* Froehlich, 1955, *I. piranga* Froehlich, 1955, and *I. potyra* Froehlich, 1958. The most notable morphological feature of *Issoca* is a cephalic glandulo-muscular organ. This organ consists of a cephalic retractor muscle derived from the ventral longitudinal cutaneous musculature, and a set of adhesive glands piercing the ventral surface of the cephalic region (Froehlich 1955). In contrast to other representatives of Geoplaninae (namely, *Choeradoplana* Graff, 1899, *Cephaloflexa* Carbayo & Leal-Zanchet, 2003, *Supramontana* Carbayo & Leal-Zanchet, 2003, and *Luteostriata* Carbayo, 2010), the cephalic retractor muscle in Issoca is typically circular in cross-section and is traversed by muscle fibers of the parenchymal subneural musculature (Froehlich 1955, Carbayo and Leal-Zanchet 2003). The retractor muscle is externally imperceptible, but the spoon-shaped cephalic region of the body helps recognize representatives of the genus. This latter feature was noted in *Choeradoplana spatulata* Graff, 1899 by Froehlich (1955), who transferred the species to *Issoca* (Froehlich, 1955). Yet the internal organs of *I. spatulata* remain unstudied.

**Figure 1.**
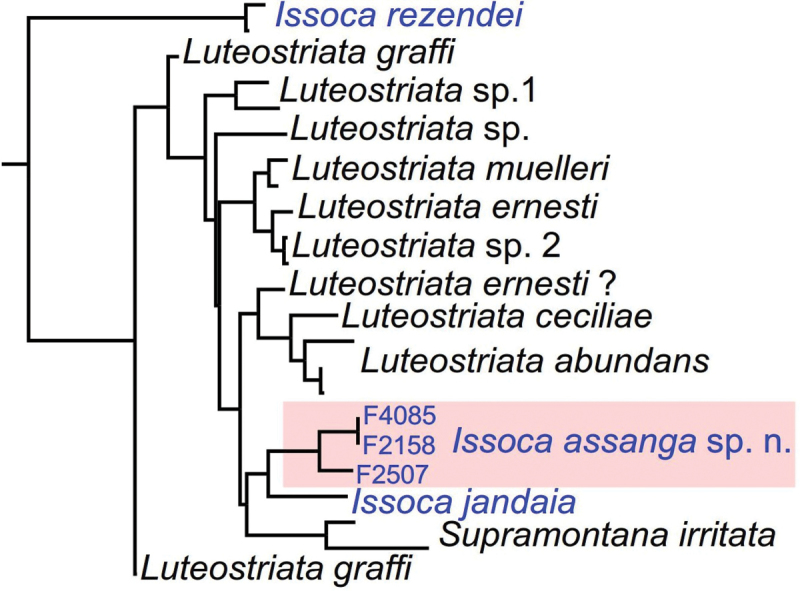
Molecular phylogenetic relationships of species of the so-called clade LIS (modified from Carbayo et al. 2013). *Issoca assanga* sp. n. was named as *Issoca* sp. 1 in Carbayo et al. (2013).

The phyletic status of *Issoca* was briefly discussed by Carbayo et al. (2013) in a paper focused on the molecular phylogeny of Geoplaninae. In that work, the genera *Issoca* (represented by three species), *Luteostriata* Carbayo, 2010 (10 species, including some polyphyletic species), and *Supramontana* Carbayo & Leal-Zanchet, 2003 (1 species), constituted the so-called clade LIS (Fig. 1). In this clade, *Issoca* turned out to be polyphyletic, with *I. rezendei* being the sister-species of all other members of the clade. Furthermore, *Luteostriata* was shown to be paraphyletic, and *Supramontana* was revealed as the sister species of a group constituted by *I. jandaia* and the undescribed species *Issoca* sp. 1. All species in the clade LIS have a cephalic retractor muscle (Froehlich 1955, 1958, Carbayo and Leal-Zanchet 2003, Carbayo 2010). No further taxonomic works were published on this genus. Herein we describe *Issoca* sp. 1 (Carbayo et al. 2013) and discuss the phyletic status of the genus from a morphological perspective.

## Materials and methods

The specimens were collected in the Atlantic Forest in the States of Espírito Santo and Rio de Janeiro, Brazil. We conducted searches on soil litter and trails during the day and night, with the light of a flashlight. Geographic coordinates of collecting sites were recorded either with Garmin eTrex GPS device (Espírito Santo) or with Googlemaps (Rio de Janeiro). Specimens were photographed, then killed with boiling water and subsequently fixed in 10% formalin. In the laboratory, the specimens were cut into pieces, dehydrated in a graded ethanol series, cleared in clove oil, then infiltrated and embedded in Paraplast® Tissue Embedding Medium. Tissue blocks were sectioned at 7 μm intervals using a microtome and affixed the sections with albumin-glycerol (1:1) onto glass slides placed on a hot plate, and stained them according to Cason (1950). The sections were dehydrated in a graded ethanol series, cleared in xylene, and mounted in synthetic balsam. The slides were examined with an optical microscope, and drawings of the copulatory apparatus were made with the aid of a camera lucida attached to the microscope. Photomicrographs were taken with a digital camera attached to the microscope and edited with GIMP (GNU Image Manipulation Program 2.8.16; The GIMP team www.gimp.org, 1995–2016). Figures of sagittal and horizontal views are orientated with anterior to the left. The type material is deposited at the *Museu de Zoologia da Universidade de São Paulo* (*MZUSP*).

### Abbreviations of figures

**af** annular-shaped fold of male atrium

**cb** cerebral ganglion

**co** common glandular ovovitelline duct

**dc** parenchymal layer of deccussate fibres

**di** diagonal parenchymal muscle

**dv** dorso-ventral parenchymal muscle

**e** eye

**ej** ejaculatory duct

**ep** esophagus

**f** fold

**fa** female atrium

**fd** female genital canal

**g** gonopore

**gl** glands

**gs** ventral glandular surface of the cephalic region

**i** intestine

**ln** normal longitudinal cutaneous muscles

**m** muscle

**ma** male genital atrium

**mc** common muscle coat

**mo** mouth

**o** ovary

**ov** ovovitelline duct

**pb** penis bulb

**po** pharyngeal pouch

**pp** penis papilla

**pv** prostatic vesicle

**r** cephalic retractor muscle

**sb** subintestinal transverse muscles

**sc** subcutaneous nerve net

**sd** sperm duct

**se** necks of secretory cells

**sg** shell glands

**sk** sunken longitudinal cutaneous muscles

**sn** subneural transverse muscles

**sp** supraintestinal transverse muscles

**t** testis

**vi** vitellaria

**vn** ventral nerve plate

## Results

### Taxonomic section

#### Family Geoplanidae Stimpson, 1857

##### Subfamily Geoplaninae Stimpson, 1857

###### Genus *Issoca* Froehlich, 1955

####### Issoca assanga

Taxon classificationAnimaliaORDOFAMILIA

sp. n.

http://zoobank.org/FD7F2CF1-B799-4215-B75C-80E16FA90B1D

######## Synonymy.

*Issoca* sp. 1; Carbayo et al. (2013).

**Type material. Parque Estadual do Desengano** (-21.87; -41.91), Santa Maria Madalena, Rio de Janeiro State, Brazil: **Holotype F4085** (MZUSP PL. 1085): J. Pedroni et al. col., 13 August 2009: sagittal sections of copulatory apparatus on 28 slides. **Paratype F4057** (MZUSP PL. 1082): J. Pedroni et al. col., 12 August 2009: sagittal sections of copulatory apparatus on 29 slides.

**Reserva Biológica Augusto Ruschi** (-19.88; -40.54), Santa Teresa, Espírito Santo State, Brazil: **Paratype F2158** (MZUSP PL 1020): F. Carbayo et al. col., 26 March 2008: fixed in 80% ethanol. **Paratype F2250** (MZUSP PL. 1023): F. Carbayo et al. col., 24 May 2008: fixed in 80% ethanol. **Paratype F2266** (MZUSP PL. 1024): F. Carbayo et al. col., 24 May 2008: fixed in 80% ethanol. **Paratype F2274** (MZUSP PL. 1025): F. Carbayo et al. col., 24 May 2008: sagittal sections of copulatory apparatus on 33 slides; transverse sections of cephalic region on 7 slides; horizontal sections of portion containing ovaries on 22 slides; sagittal sections of pharynx region on 34 slides; transverse sections of pre-pharyngeal region on 8 slides. **Paratype F2309** (MZUSP PL. 1032): F. Carbayo et al. col., 24 May 2008: fixed in 80% ethanol. **Paratype F2394** (MZUSP PL. 1037): F. Carbayo et al. col., 27 May 2008: sagittal sections of copulatory apparatus on 18 slides; sagittal sections of pharynx region on 20 slides; transverse sections of cephalic region on 4 slides; horizontal sections of portion containing ovaries on 8 slides; and transverse sections of pre-pharyngeal region on 6 slides. **Paratype F2470** (MZUSP PL. 1042): F. Carbayo et al. col., 29 May 2008: transverse sections of cephalic region on 14 slides; horizontal sections of portion containing ovaries on 48 slides; sagittal sections of a portion posterior to ovaries on 23 slides; sections immediately before pre-pharyngeal region on 33 slides; transverse sections of pre-pharyngeal region on 16 slides; sagittal sections of pharynx region on 41 slides; sagittal sections of copulatory apparatus on 61 slides. **Paratype F2507** (MZUSP PL. 1045): F. Carbayo et al. col., 29 May 2008: preserved in 80% ethanol.

######## Type locality.

Parque Estadual do Desengano, Santa Maria Madalena, Rio de Janeiro State, Brazil.

######## Diagnosis.

Species of *Issoca* up to 97 mm in length. Widest dorsal pigment bands with 33–40% of body width. A few cutaneous muscle bundles in-sunk in the pre-pharyngeal region. Cephalic retractor muscle and the sub-neural parenchymal muscle not intersecting with each other. Copulatory apparatus relatively long. Prostatic vesicle extrabulbar, proximally dilated, and non-anastomosed. Ejaculatory duct thin, with its opening at the tip of the penis papilla; this papilla is conical with dorsal insertion posterior to the ventral one. Female atrium spacious with some lateral folds. Common glandular duct almost as long as the female atrium. Common muscle coat envelops male and female atria.

######## External morphology.

**Figure 2.**
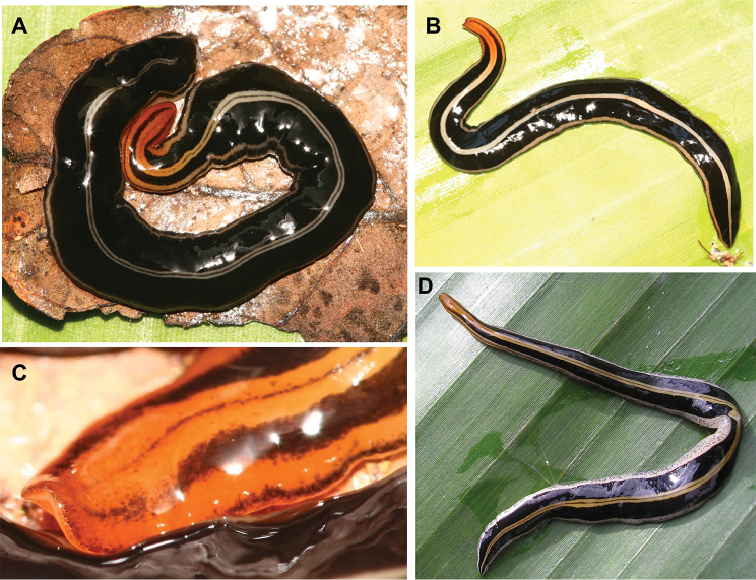
*Issoca assanga* sp. n. Living specimens. Cephalic region is orangish. **A** paratype F2266 **B** paratype F2309 **C** Cephalic region of paratype F2250 **D** paratype F4085. Scale bars not available.

The body is elongated with nearly parallel margins (Fig. 2A–B). Its cephalic region (5% of the body length) is sometimes slightly laterally dilated before converging to the rounded anterior extremity of the body, which is slightly concave ventrally (Fig. 2C). The posterior extremity is pointed. The dorsum is slightly convex, the ventral side is flattened, and the body margins are rounded so that the section of the body is elliptic. Six mature, fixed specimens measured 48–97 mm in length. Fixed, paratype F2470 is 71 mm in length, 7 mm in width and 1.7 mm in height. The creeping sole is as wide as 74–84% of body width at the pre-pharyngeal region (paratypes F2470 and F2394, respectively). The mouth lies at a distance from the anterior extremity equal to 55% of body length; the gonopore is at 80% (paratype F2274).

**Figure 3.**
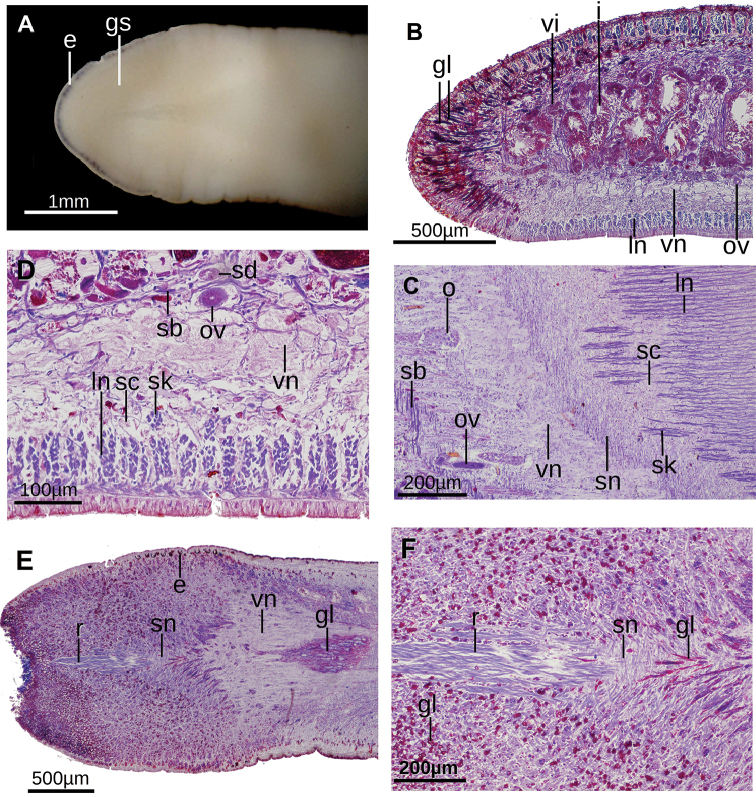
*Issoca assanga* sp. n. **A** Ventral view of cephalic region of fixed paratype F2274. (B-L): Photomicrograph of histological sections **B** transverse section of left side of pre-pharyngeal region of paratype F2394 **C** Horizontal section of paratype F2394 at the level of the ovaries **D** transverse section of prepharyngeal region of paratype F2394 **E, F** horizontal section of cephalic region of paratype F2394.

The dorsal color consists of a cream-colored (specimens from Ruschi, Fig. 2A–B) or yellowish (specimens from Desengano, Fig. 2D) median stripe (6.7% of body width). This stripe is longitudinally divided by a very thin black median line (2.2%), which can be very tenuous and discontinuous (Fig. 2B). The .median stripe is bordered on either side by a wide black band (33–40%). This wide band is externally bordered by a whitish stripe (4–6.7%). The median and the lateral stripes gradually pass into orange of cephalic region (Fig. 2C). A marginal zone is either pigmented with a black stripe (5%, specimens from Ruschi, Fig. 2A–C) or mottled with black pigment spots (8.3%; Desengano, Fig. 2D). The ventral side is whitish, passing into orange of the cephalic surface. This surface (5% of body length) extends along the margins of the body progressively occupying a wider surface towards the anterior extremity until they fuse at 1 millimeter from the anterior extremity of the body (Fig. 3A).

The eyes are formed by one pigmented cup 50 µm in diameter. They contour the anterior extremity in a row of 2–3 eyes along the first 2 millimeters (Fig. 2C); going backwards, they spread progressively on each side of the dorsum in a band with 33% of the body width until the end of anterior half of the body. Posterior to this region they are scarcer and the band narrows until posterior extremity of the body.

######## Internal morphology.

The sensory pits are 30 µm deep and are distributed in a simple ventro-lateral row, from the very anterior extremity up to at least 38% of body length. In the prepharyngeal region (Fig. 3B), rhabditogen cells and cell glands producing erythrophil granules open through the dorsal epithelium (Fig. 3B); necks of these glands are thick - 20 µm in width. Three additional gland types discharge their content through dorsal and marginal epidermis. They become progressively more abundant from the midbody towards the body margins. These gland cells are as follows: one type of cell with cell neck 20 µm in diameter produces xanthophilic granules; the second type produces xanthophilic granules, and its neck is 8–10 µm in diameter; the third type produces cyanophilic granules and its neck is 8–10 µm thick. The ventral epidermis is pierced by two types of scarce cell glands, each of them secreting either erythrophilic or xanthophilic granules.

The cutaneous musculature comprises the three typical layers of Geoplaninae, i.e., a subepithelial circular layer followed by a double diagonal layer with decussate fibers, and a strongly developed longitudinal layer, 60–125 µm thick. The fibers of the latter muscle layer are gathered into compact bundles (Fig. 3B–D). A few small muscle bundles of the cutaneous longitudinal muscle are sunken in the pre-pharyngeal region (Fig. 3C–D). The number of these sunken muscle fibers increases towards the cephalic region. The cutaneous musculature thickness relative to body height at the pre-pharyngeal region is 16–18%.

The three usual parenchymal muscle layers are present throughout the body: a dorsal layer of diagonal decussate fibers (50 μm thick, or 5% of the body height, paratype F2470), a transverse supraintestinal layer (40 μm, or 4%), and a transverse subintestinal layer (50 μm, or 5%). Additionally, there is a subneural layer (40 μm, or 4%) of transverse muscles. Dorso-ventrally oriented parenchymal fibers are abundant in the pre-pharyngeal region.

**Figure 4.**
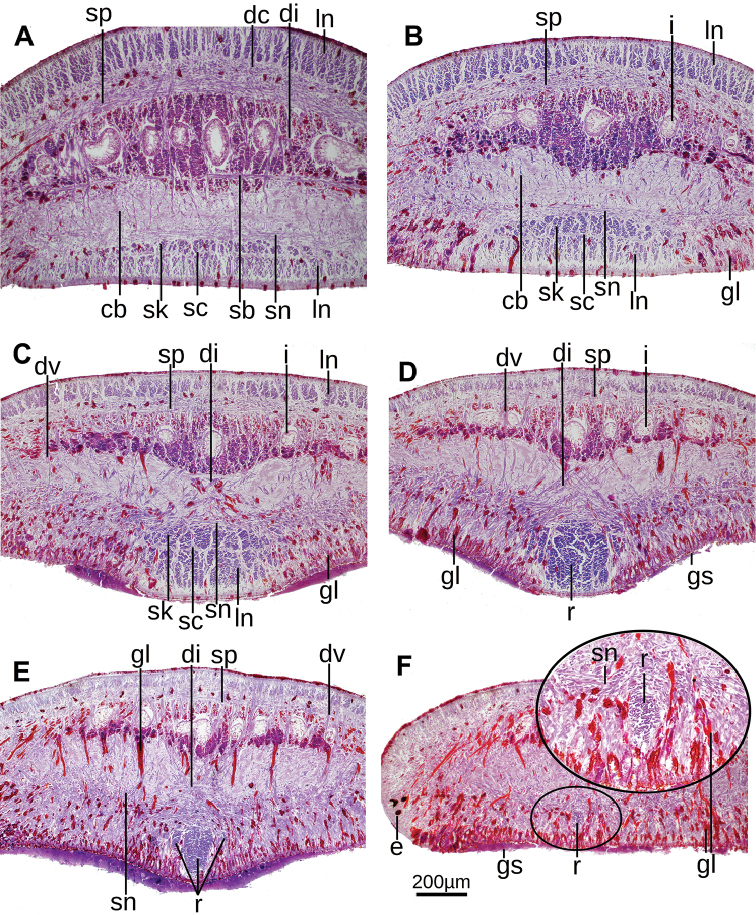
Serial transverse sections of the cephalic region of paratype F2470 at 4.0, 2.6, 2.0, 1.6, 1.3, and 1.0 mm from anterior extremity, respectively. Encircled region in **F** is enlarged in the inset. **A–E** at the same magnification as **F**.

The muscular organization changes (Fig. 4) in the anterior region of the body with respect to that of the pre-pharyngeal region. The number of sunken ventral longitudinal cutaneous fibers increases at 5 millimeters from the anterior extremity of the body (equal to 10% of body length in paratype F2394), and bundles of both normal and sunken ventral cutaneous muscles concentrate medially to give rise to the cephalic retractor muscle. The retractor is lens-shaped in cross-section at 4–2.6 mm from anterior extremity (Fig. 4A–B). It becomes elliptic at 2.0 mm (Fig. 4C) and roughly quadrangular (Fig. 4D) at 1.6 mm from anterior extremity. Muscle fibers of the retractor muscle are gathered in few but thick bundles. From this region towards anterior extremity, fibers of the retractor muscle progressively detach in bundles that run to the body sides (Fig. 4D–F). As they detach, the retractor muscle becomes less apparent until it disappears close to the anteriormost extremity of the body. It could not be determined whether other fibers from this muscle run dorsally. Before disappearing the retractor muscle is elliptic in cross section (Fig. 4E–F).

In the cephalic region, diagonal, supraintestinal, and subneural parenchymal muscle layers are apparent and placed in the same position relative to the cutaneous longitudinal muscles. Even the subneural muscle and its fibers continue running over the retractor muscle (Fig. 4). The subintestinal parenchymal muscle layer is less apparent, and this layer is the first to disappear as it approaches the anterior extremity of the body. All parenchymal muscles fade out at the anteriormost body portion.

Dorso-ventral parenchymal muscle fibers are more abundant in the cephalic region than on the rest of the body, and they are frequently gathered in bundles of 3–10 fibers each. These fibers run approximately dorso-ventrally, connecting dorsal epidermis with the ventral glandular epidermis. Medially, these fibers run obliquely from the dorsal epidermis of one side of the body to anchor to the ventral glandular epidermis of the other body side, thereby rimming the retractor muscle (Fig. 4C–E).

The glandular surface of the ventral epidermis, orangish in color in living animals, widens towards anterior extremity of the body as the muscle fibers of the retractor concentrate medially. This surface is incompletely bipartite (Fig. 3A) and is richly pierced by gland cells (with 10–12 μm thick necks) producing erythrophilic granules, and by scarce glands (with 12–18 μm thick necks) producing cyanophilic granules.

**Figure 5.**
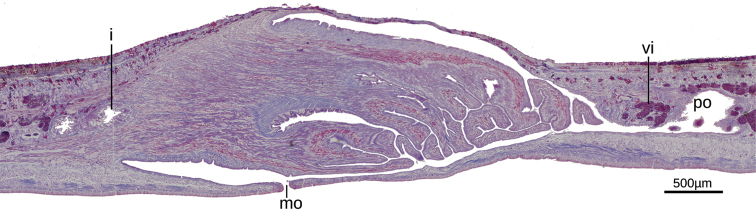
*Issoca assanga* sp. n. Photomicrograph a sagittal section of the pharynx of paratype F2394.

The mouth is situated at a distance from the root of the pharynx equivalent to 25% of pharyngeal pocket length (Fig. 5). An esophagus is present with 13–20% of pharynx length. The pharynx is cylindrical, with dorsal insertion posterior to the ventral and located at mouth level. The lining epithelium of the pharyngeal pouch is squamous, non-ciliated, surrounded by a simple layer of circular fibers, followed by a layer of diagonal fibers (10 µm thick). The outer and inner pharyngeal epithelia are flat and ciliated. The outer epithelium is underlain by a longitudinal muscle (2.5 µm thick) followed by a circular muscle (17–75 µm), the latter with some longitudinal fibers interspersed. The inner epithelium is surrounded by a circular muscle (45–90 µm), followed by a longitudinal muscle (25 µm).

The central nervous system mainly consists of a ventral nerve plate. Cerebral ganglia (Fig. 4B) extend along the body from 1 millimeter to 4 millimeters behind the anterior tip (2% and 8%, respectively, paratype F2394).

**Figure 6.**
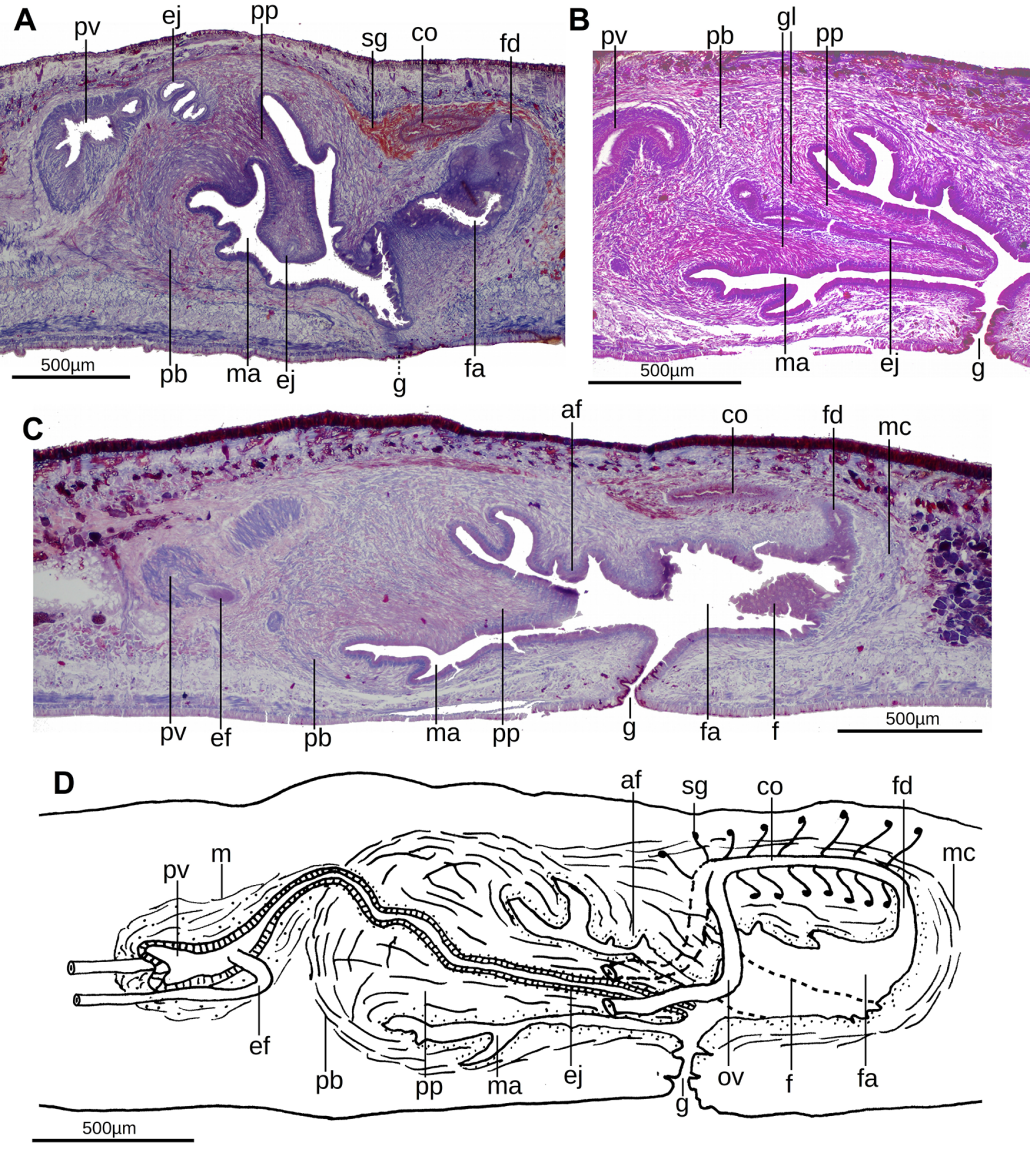
*Issoca assanga* sp. n. **A** photomicrograph of a sagittal section of the copulatory apparatus of paratype F2470 **B** photomicrograph of a sagittal section of the penis papilla of paratype F2394 **C** photomicrograph of a sagittal section of the copulatory apparatus of paratype F2394 **D** diagrammatic representation of the copulatory apparatus of paratype F2394 from sagittal sections.

The testes are located under the supraintestinal transverse muscle layer, partially between the intestinal diverticula. They extend from the level of the ovaries to nearly the root of the pharynx. The sperm ducts run between the subintestinal parenchymal muscle layer, dorsally to the ovovitelline ducts. They open into the antero-lateral portion of the prostatic vesicle. This vesicle is extrabulbar and proximally dilated, curves dorsally and penetrates the dorso-anterior aspect of the penis bulb. This vesicle is lined with a ciliated, columnar epithelium, showing an irregular free surface in its anterior portion. The prostatic vesicle receives fine erythrophilic granular secretions derived from glands in the parenchyma. These penetrate the ciliated, columnar epithelium lining of the vesicle to discharge into the lumen. The vesicle is surrounded by intermingled decussate, circular, and longitudinal fibers. The prostatic vesicle passes into the relatively thin ejaculatory duct that is lined by a ciliated cuboidal epithelium surrounded by circular muscle. The ejaculatory duct is proximally sinuous and distally straight through the mid penis bulb, terminating at the tip of the penis papilla. The ejaculatory duct is lined with a cuboidal, ciliated epithelium and is surrounded by a circular muscle. The protrusible penis papilla is conical, slightly inclined ventrally, and with its dorsal insertion posterior to the ventral insertion (Fig. 6A–D). The penis papilla is as long as the male atrium, and is lined with a cuboidal, non-ciliated epithelium, and is surrounded by a circular muscle followed by a longitudinal muscle; some fibers of both muscles are intermingled. Numerous secretory cells located in the adjacent parenchyma produce erythrophilic granules (Fig. 6B) that are discharged along the length of the papilla.

The male atrium is mostly occupied by the penis papilla. It is more spacious in its anterior portion than in the posterior, and shows folded walls. One of these folds is a transverse, annular-shaped fold located halfway of the atrial length. From the roof of the distal portion of the male atrium, a large fold projects laterally and continues along the female atrium. The male atrium is lined with a columnar, non-ciliated epithelium, and is pierced by gland cells producing erythrophilic granules. A circular muscularis (5 µm thick) encircles the male atrium.

The ovaries are 500 µm long in the antero-posterior body axis and 200 µm wide. They are located immediately above the ventral nerve plate, at a distance from anterior tip equivalent to 26% of body length (Fig. 3D). The ovovitelline ducts arise from the dorso-external surface of the anterior portion of the ovaries, and run backwards above the ventral nerve plate. They ascend laterally to the gonopore region, and subsequently unite dorsally to form a common ovovitelline duct just dorsal to the anterior section of the female atrium (Fig. 6D). The distal third of the ascending portion of these paired ducts receives shell glands. The ovovitelline ducts unite to form the common glandular ovovitelline duct, which runs caudally and progressively curving to the ventral side to communicate with the female genital canal. This canal is a projection of the postero-dorsal portion of the female atrium that runs dorsally and slightly anteriorly. The female atrium is an irregular, spacious cavity. Its walls are partially projected into its lumen. One of these folds is continuous with a fold coming from the male atrium (Fig. 6D).

**Figure 7.**
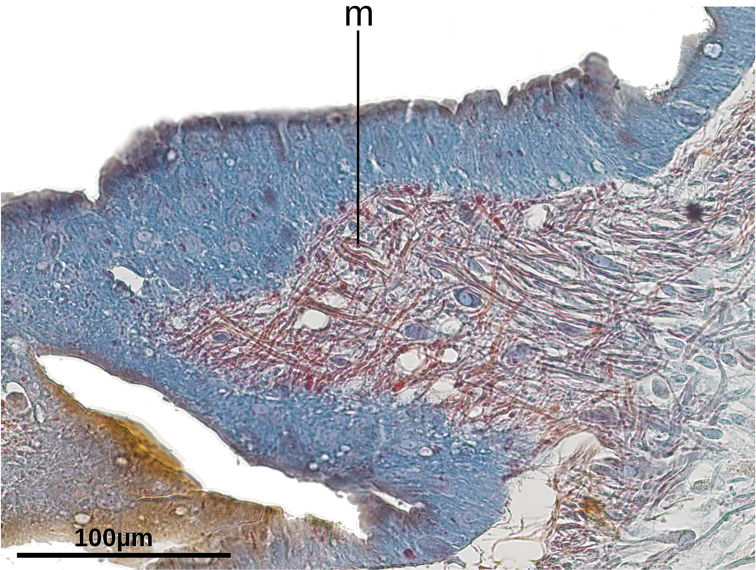
*Issoca assanga* sp. n. Photomicrograph of a sagittal section of the lining epithelium of the posterior section of the female atrium of the holotype.

The female atrium is lined with a columnar epithelium, which is lacunar in aspect in some parts. In the anterior portion of the female atrium, the simple columnar epithelium is 25 μm high; whereas the posterior portion is lined by a pseudostratified columnar epithelium (Fig. 7). The muscularis of the female atrium consists of two muscle layers; a simple (2.5 µm thick) longitudinal muscle followed by a circular muscle (10 µm), both partially intermingled. The female atrium receives gland cells producing erythrophilic granules. The male atrium is 1.2 times longer than the female. The common muscle coat is well-developed, and wraps the male and female atria.

######## Etymology.

The specific epithet refers to the Tupi (indigenous Brazilian tribe) name *assanga*, meaning dense, thick (Tibiriçá 1984). It refers to the apparent cephalic glandulo-muscular organ of the new species.

######## Distribution.

Parque Estadual do Desengano (Municipality of Santa Maria Madalena, Rio de Janeiro State) and Reserva Biológica Augusto Ruschi (Municipality of Santa Teresa, Espírito Santo State), Brazil.

######## Discussion.

The external aspect of *I. assanga* allows one to readily distinguish it from the remaining species of the genus by being considerably larger (97 mm in length after fixation) than the largest previously described species, i.e. *I. jandaia*, which is 50 mm in length alive, and 32 mm after fixation. Although the chromatic pattern of all species of the genus is striped, the stripes are as wide as 33–40% of the body width only in *I. assanga* sp. n. The widest colored stripe in other species of the genus is found in *I. potyra*, with a pair or paramedian bands with 17% of the body width each (Froehlich 1955, 1958; Graff 1899; Marcus 1951).

Regarding the digestive system anatomy, the new species can be distinguished from the other species because the mouth is situated at a distance from the root of the pharynx equivalent to 25% of pharyngeal pocket, whereas the mouth in the other species is at a distance equal to 33% (*I. jandaia*) or >30% (*I. rezendei* and *I. piranga*, 50%; *I. potyra*, 67%; Froehlich 1955, 1958; Marcus 1951).

The new species can also be readily distinguished from *I. rezendei* by the general shape of the copulatory apparatus. Unlike the new species (with a relatively long copulatory apparatus, an extrabulbar prostatic vesicle, a horizontal penis papilla and a conspicuous female atrium), the copulatory apparatus in *I. rezendei* is relatively compact, the prostatic vesicle is intrabulbar, the penis papilla is vertical, and the female atrium is absent (Marcus 1951). The outlines of the copulatory apparatuses of the remaining species of *Issoca* are comparable to that of the new species: those being a relatively long and extrabulbar prostatic vesicle; and a penis papilla, or a papilla-like fold horizontal or moderately inclined (Froehlich 1955, 1958). However, the three species *I. jandaia*, *I. piranga* and *I. potyra* (*I. spatulata* is not in this comparison because its copulatory apparatus is yet unknown), differ from the new species in the following details. In contrast to *I. assanga* sp. n., the ejaculatory duct of *I. jandaia* opens into a cavity inside a penis papilla-like fold of the male atrium, the common glandular duct is relatively long, and the female atrium is intensely folded. The penis papilla of *Issoca piranga* occupies only the anterior half of the male atrium, and the muscle coat enveloping the male atrium is separated from that wrapping the female one (Froehlich 1955), whereas in the new species the penis papilla is as long as the male atrium, and there is a common muscle coat wrapping male and female atria. Finally, *I. potyra* differs from the new species in that the wall of the prostatic vesicle is anastomosed, the ejaculatory duct is wide and irregular and it opens into the ventral side of the penis papilla, the male atrium is separated from the female one by a fold which is richly pierced by cyanophilic glands, the common glandular oviduct is relatively short, and the muscle coat enveloping the male atrium is separated from that of the female one (Froehlich 1958), whereas in *I. assanga* sp. n., there are no anastomoses in the prostatic vesicle, the ejaculatory duct is relatively thin, and terminates at the tip of the penis papilla, the male atrial fold is not pierced by cyanophilic glands, the common glandular oviduct is relatively long, and the muscle coat wraps both male and female atria.

One diagnostic feature of the genus is that the cephalic retractor muscle and the subneural parenchymal muscle are intersected, a condition present in the type species of the genus *Issoca rezendei* (see diagnosis in Carbayo and Leal-Zanchet 2003). This feature is the only mismatching diagnostic trait of *I. assanga* sp. n. This condition could be polymorphic within the genus. However, two aspects support a different interpretation: (a) the polyphyly of *Issoca* has been highlighted (Carbayo et al. 2013); and (b) the general anatomy of the copulatory apparatus of *I. rezendei* is very different from that of the remaining species of the genus. These points suggest that rather than an interspecifically polymorphic character, the muscle intersection might reflect the polyphyletic status of the genus (see Fig. 1). Description of the muscular cephalic retractor muscle in the other species of *Issoca* is limited. The muscular organization of the glandulo-muscular organ in *I. jandaia* was reported by Froehlich (1955) as following the same structure as *I. rezendei*. The glandulo-muscular organ in *I. piranga* was described as being very similar to that of *I. jandaia* (see Froehlich 1955). Froehlich (1958) mentioned that the glandulo-muscular organ (i.e., the cephalic retractor muscle plus associated viscid glands) in *I. potyra* “is similar to that of the other species of the genus”. However, his diagrammatic reconstruction of the organ shows the cephalic retractor muscle underneath the subneural muscle layer, not intersected. Moreover, the retractor in *I. rezendei* diminishes by means of separating its fibers towards the body margins and the back (Carbayo and Leal-Zanchet 2003), whereas the fibers in *I. assanga* were observed only running to the body margins. This lack of morphological details reinforces the need of a taxonomic revision of the clade LIS, as already suggested by Carbayo et al. (2013).

## Supplementary Material

XML Treatment for Issoca assanga
